# Trends in Operative Fixation of Olecranon Fractures in Elderly Australians: A Nationwide Study From 2000 to 2024

**DOI:** 10.7759/cureus.103453

**Published:** 2026-02-12

**Authors:** Christopha J Knee, Vincent An, Ryan J Campbell, Venkatesha Venkatesha, Brahman Sivakumar, Cameron Handford, Michael Symes

**Affiliations:** 1 Department of Orthopaedics and Trauma Surgery, Royal North Shore Hospital, Sydney, AUS; 2 Research Directorate, Northern Sydney Local Health District, Royal North Shore Hospital, Sydney, AUS; 3 Department of Hand and Peripheral Nerve Surgery, Royal North Shore Hospital, Sydney, AUS; 4 Department of Orthopaedics and Trauma Surgery, Royal North Shore Hospital, Sydney, Sydney, AUS; 5 Department of Orthopaedic Surgery, St George and Sutherland Hospitals, Sydney, AUS

**Keywords:** elbow fracture, elderly, epidemiology, olecranon fractures, operative fixation, surgery, trends

## Abstract

Background: Olecranon fractures are a common fragility injury in elderly patients, typically resulting from low-energy mechanisms. While evidence supporting nonoperative management in this population has increased, rates and long-term trends in operative fixation of olecranon fractures in elderly Australians remain poorly characterised. This study evaluated national trends in operative management of olecranon fractures in Australians aged 75 years and older from 2000 to 2024.

Methods: A retrospective analysis of Medicare Benefits Schedule data was performed from 2000 to 2024. Olecranon fracture fixations were identified using item number 47399 (treatment of olecranon fracture by open reduction). Patients aged 75 years and older were included. Annual case volumes and per-capita rates were stratified by year and sex. Temporal trends were assessed using linear regression and correlation analysis.

Results: A total of 3,644 olecranon fixation procedures were performed in patients aged 75 years and older. Annual volumes increased over time, reaching 193 procedures in 2024, with a mean annual increase of 3.9 cases (p < 0.001). Female patients accounted for 82.7% of all procedures, though their proportional representation declined 0.45% per year (p < 0.001). Male per-capita fixation rates increased 2.2-fold (r = 0.57; p < 0.01), while female rates remained stable.

Conclusion: Operative fixation of olecranon fractures in Australians aged 75 years and older increased over the 25-year study period, despite growing evidence supporting nonoperative management in this population. Although female patients accounted for the majority of procedures, their per-capita fixation rates remained stable, while male rates doubled. These findings reflect evolving surgical practice and support further research into patient selection, clinical outcomes and economic implications.

## Introduction

Olecranon fractures comprise approximately 10% of upper limb fractures and represent a common fragility injury in the elderly [1-4]. Most of these injuries result from low-energy mechanisms, typically a fall from standing height [3,4]. Displaced olecranon fractures have traditionally been managed operatively via tension band wiring (TBW) or plate fixation (PF) [5-7]. While both methods yield satisfactory union and functional outcomes in elderly patients, complication and reoperation rates remain high and have been reported to exceed 50% in some studies [4,5,8,9].

In light of these risks, nonoperative management has received increasing attention and utilisation in elderly patients, particularly those over 75 years of age [6,10,11]. A growing body of evidence evaluating nonoperative treatment has reported low complication rates, good functional outcomes and high patient satisfaction [2,4,5,9]. A recent meta-analysis found that despite low radiographic union rates, most elderly patients achieved excellent functional outcomes following nonoperative treatment [9]. Nonetheless, a recent survey of 197 orthopaedic surgeons revealed that the majority still preferred operative management for both simple and comminuted olecranon fractures in patients over 75 years of age [12].

The burden of olecranon fractures is expected to increase with population ageing. Despite growing interest in the optimal management of elderly olecranon fractures, limited data exist regarding contemporary trends in operative treatment, both in Australia and internationally. Characterising these trends is important to inform clinical decision-making, health policy and healthcare resource planning. This study aims to assess temporal trends in the operative management of olecranon fractures in Australians aged 75 years and older, using Medicare Benefits Schedule (MBS) data from 2000 to 2024.

## Materials and methods

This study utilised publicly available data from the MBS, administered by the Australian Government Department of Health. The MBS outlines subsidised medical services through designated item numbers. As the dataset is de-identified and publicly accessible, and contains no personal or direct patient information, formal ethics approval was not required.

Procedural data were obtained for item number 47399 (treatment of fracture of the olecranon, by open reduction) [13]. Procedures performed in patients aged 75 years and older, consistent with the MBS registry age stratification, were extracted for the period from 2000 to 2024. This extended study period was selected to capture long-term changes in operative management, including shifts associated with the emergence of evidence supporting nonoperative treatment, as well as broader temporal changes in operative utilisation. Data were stratified by calendar year and sex.

Per-capita rates (services per 100,000 population) were obtained directly from the Medicare Item Reports provided by Services Australia [14]. These rates are calculated by dividing the number of services processed in a given month by the number of individuals enrolled in Medicare at the end of that month, and are aggregated by calendar year.

Descriptive statistics were used to summarise annual case volume and overall trends [15]. Pearson correlation coefficients were calculated to assess the linear association between calendar year and annual per-capita rates, stratified by sex. Linear regression analyses were used to estimate annual changes in procedure volume. The assumption of no serial correlation was assessed using the Breusch-Godfrey Lagrange multiplier (LM) test. Where this assumption was violated, the model specification was adjusted to include autoregressive components. To evaluate relative changes over time, Poisson regression models were fitted, and results were reported as incidence rate ratios (IRRs) with corresponding 95% confidence intervals (CIs).

All statistical analyses were conducted using jamovi (version 2.6.5; The jamovi project, Sydney, Australia) and Stata (version 18.0; StataCorp LLC, College Station, TX). Figures were generated using Microsoft Excel (version 16.77; Microsoft Corporation, Redmond, WA).

## Results

**Figure 1:**
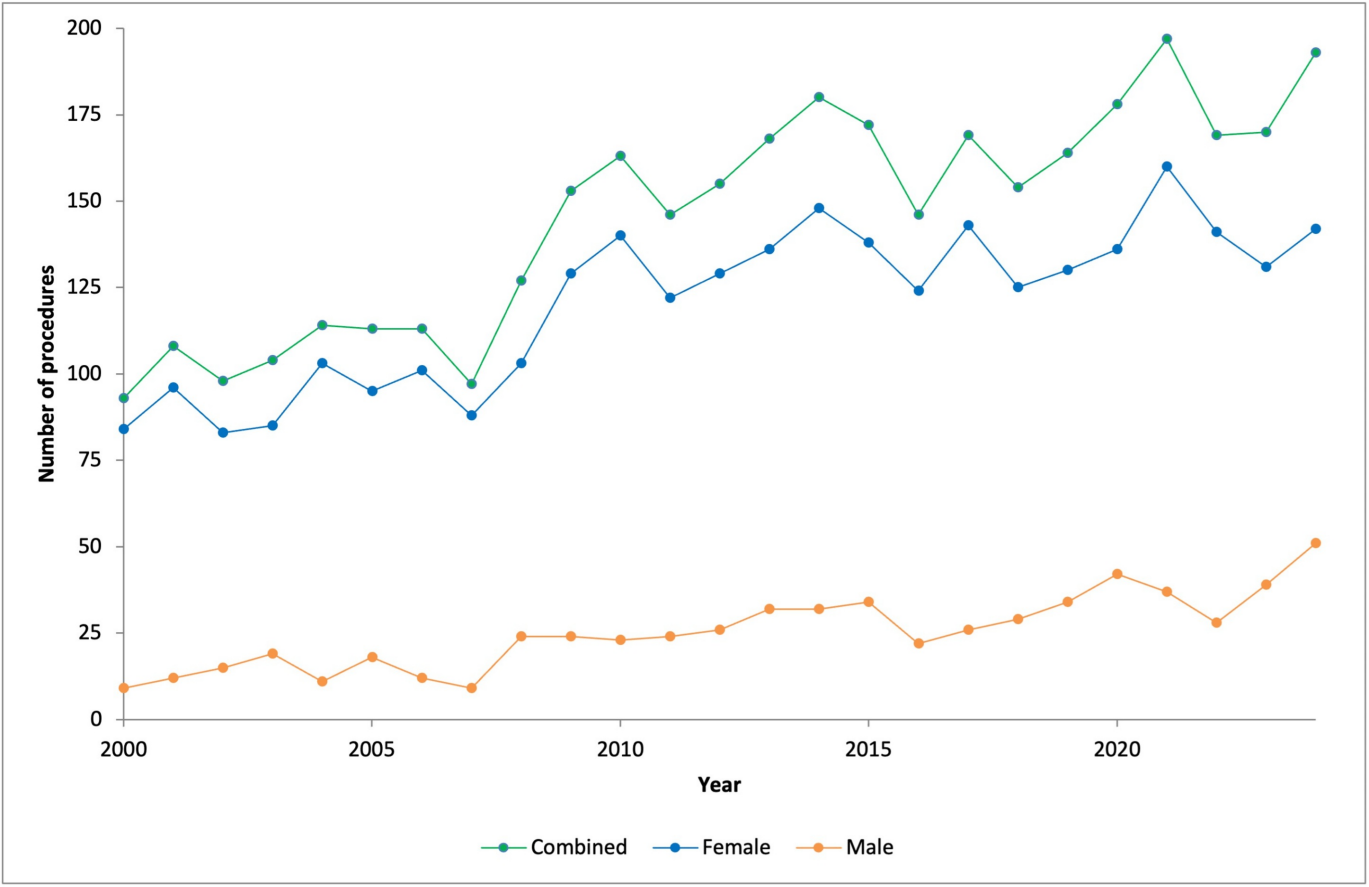
Annual number of operative fixation procedures for olecranon fractures in patients aged 75 years and older

A total of 3,644 olecranon fracture operative fixation procedures were performed in Australians aged 75 years or older from 2000 to 2024. The mean annual case volume was 145.8 procedures (range: 93-197 per year). Annual procedural volume increased 2.08-fold over the study period, from 93 in 2000 to 193 in 2024 (Figure 1).

**Figure 2:**
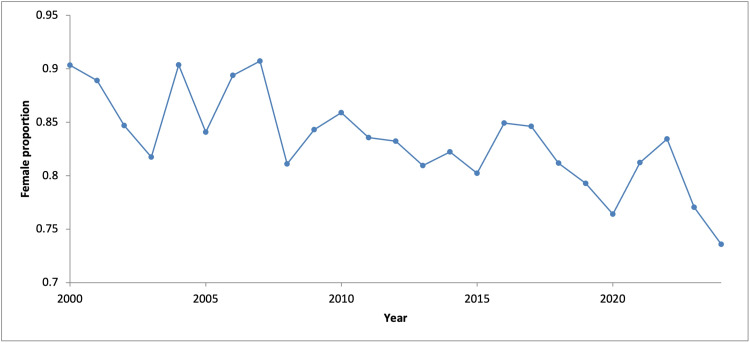
Annual proportion of female patients undergoing operative fixation for olecranon fractures

**Figure 3:**
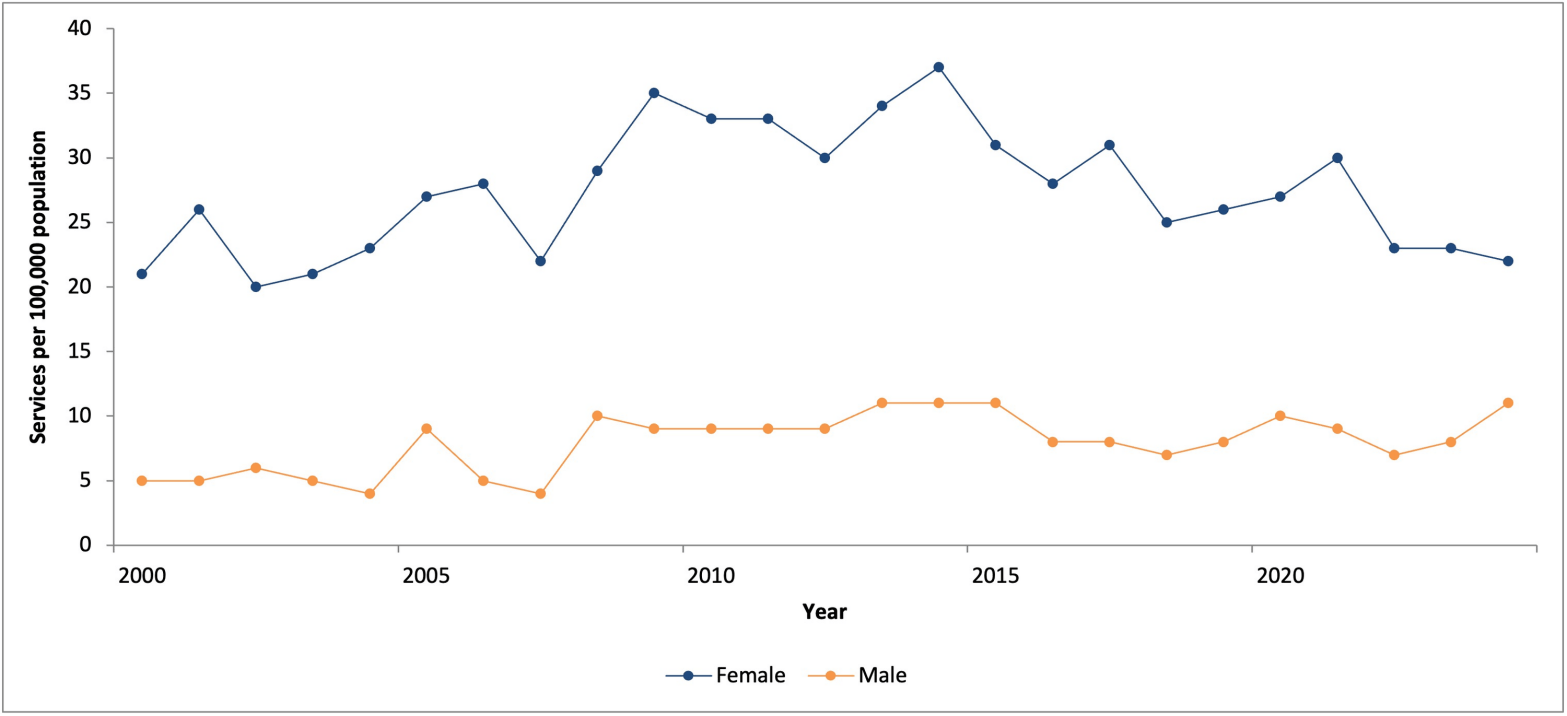
Annual operative fixation rates per 100,000 population in individuals aged 75 years and older

Female patients accounted for 82.7% (n = 3,012) of all procedures performed. However, the annual proportion of procedures performed in female patients declined by 0.45% per year (95% CI: -0.62 to -0.27, p < 0.001; R² = 0.55) (Figure 2). Per-capita analysis demonstrated a stable rate of operative fixation among female patients (r = 0.16, p = 0.451), whereas male patients demonstrated an upward trend over time (r = 0.57, p < 0.01), with per-capita rates increasing 2.2-fold over the study period (Figure 3). These correlations represent temporal associations.

Linear regression analysis demonstrated a significant increase in procedural volume over time for the total cohort, with an annual increase of 3.92 procedures (95% CI: 3.08-4.76, p < 0.001; R² = 0.80). No serial correlation was identified on Breusch-Godfrey LM testing (p > 0.05). Poisson regression analysis indicated this absolute change corresponded to a mean annual increase of 2.7% in procedural volume (IRR = 1.027, 95% CI: 1.023-1.032). Subgroup analysis demonstrated an annual increase of 2.62 procedures among females (95% CI: 1.90-3.34, p < 0.001; R² = 0.71) and 1.30 procedures among male patients (95% CI: 1.00-1.60, p < 0.001; R² = 0.77).

## Discussion

This study provides the first national, population-level analysis of trends in the operative fixation of olecranon fractures in elderly Australians. Between 2000 and 2024, the use of operative fixation in individuals aged 75 years and older more than doubled, with a steady annual increase observed throughout the study period. While female patients accounted for most procedures, their proportional representation declined over time. Notably, male per-capita fixation rates increased 2.2-fold, reflecting a substantial rise in operative management in this group.

Previous epidemiological research has demonstrated a significant increase in the incidence of olecranon fractures in patients aged 75 years and older, with a concurrent trend toward nonoperative management [16]. However, there remains a paucity of data describing longitudinal treatment patterns in this population. Historically, displaced olecranon fractures in elderly patients have been treated operatively with TBW or PF, both of which achieve union rates approaching 94% [9]. Despite these favourable union rates, operative fixation carries a substantial burden of morbidity, including reoperation, wound breakdown, infection and symptomatic hardware requiring removal; indeed, complications have been reported in up to 50% of cases [4,5,8,9].

In light of these concerns, increasing attention has been directed toward nonoperative management, with many authors advocating its use in select elderly patients. Chen et al. reported that up to 86% of elderly olecranon fractures treated nonoperatively resulted in nonunion [9]. The majority of these nonunions were asymptomatic, with most patients regaining satisfactory elbow function, including active range of motion, extension strength and the ability to perform activities of daily living [4,5,9,17]. Duckworth et al. reported a 91% satisfaction rate at two-year follow-up in low-demand elderly patients managed nonoperatively, with no patients requiring subsequent surgical intervention [7]. In addition to achieving satisfactory functional outcomes, nonoperative management has been shown to be more cost-effective, reducing healthcare expenditure, an important consideration as costs continue to rise with an ageing population [18,19].

To our knowledge, only two randomised controlled trials have directly compared operative and nonoperative management in patients aged 75 years or older. Duckworth et al. terminated their trial early due to a significantly higher rate of complications in the surgical arm, most commonly involving loss of reduction or hardware-related problems [6]. Despite early termination, no significant differences in patient-reported or clinician-assessed outcomes were observed at any time point during the first postoperative year.

More recently, Joshi et al. reported the findings of a multicentre prospective pragmatic randomised controlled trial, noting no difference in Disabilities of the Arm, Shoulder and Hand (DASH) scores or complications at 12 months between the groups [10,20]. However, the operative group achieved significantly better DASH and Mayo Elbow Performance Scores at three months, indicating a potential short-term benefit for those prioritising early recovery. Such benefits may be most relevant for elderly individuals who remain in the workforce, lead active and independent lives or have caregiving responsibilities [19]. These factors, rather than age alone, likely influence treatment decisions and may partly explain the greater uptake of operative fixation among elderly males in our study.

Our findings are consistent with recent large-scale epidemiological studies showing increasing fracture incidence among elderly male patients, while rates in female patients have remained stable [21]. The unchanged incidence in female patients may reflect the cumulative benefits of established prevention strategies, such as bisphosphonate therapy, vitamin D supplementation and hormone replacement, which are more commonly targeted towards female patients due to reduced bone mineral density and strength [21]. Conversely, the increasing fracture rates in male patients may suggest underutilisation of such measures and a need for targeted prevention [21]. Furthermore, male patients were more likely than female patients to sustain fractures while participating in sports or recreational activities, highlighting the role of activity level and functional status in injury risk, and suggesting another potential avenue for targeted education and risk-reduction strategies [21].

This study has several limitations inherent to the use of MBS data. The MBS primarily captures procedures performed on privately insured patients, whether treated in public or private hospitals, and excludes procedures funded directly by state governments for public patients. However, given the stable proportion of privately insured individuals over time, the findings are generalisable to the broader population [22-29]. Observed trends may reflect characteristics of the Australian healthcare system and may not be directly generalisable to healthcare systems that are exclusively publicly funded. Additionally, the relevant MBS item number encompasses all fixation techniques, including tension-band wiring, plating and suture band fixation, preventing differentiation between these surgical techniques. The database also only accounts for patients who underwent operative management; as a result, the proportion of elderly patients managed without surgery could not be assessed. Although the COVID-19 pandemic may have temporarily affected surgical capacity during parts of the study period, no sustained deviation from the long-term temporal trend in operative fixation was observed.

## Conclusions

This study demonstrates an increase in operative fixation of olecranon fractures among Australians aged 75 years and older over the 25-year study period. Although the majority of procedures were performed in female patients, their proportional representation declined over time, alongside rising per-capita fixation rates in male patients. These findings reflect shifts in surgical practice within this population and highlight the need for further research into clinical outcomes, optimal patient selection and the economic impact of operative treatment. With an ageing population, the management of olecranon fractures in elderly Australians is likely to place increasing demands on healthcare resources.
